# Cerebral venous thrombosis in post-lumbar puncture intracranial hypotension: case report and review of literature

**DOI:** 10.12688/f1000research.3-41.v1

**Published:** 2014-02-11

**Authors:** Mahesh P. Kate, Bejoy Thomas, P.N. Sylaja

**Affiliations:** 1 Department of Medicine, University of Alberta, Edmonton, T6G0T1, Canada; 2 Department of Imaging Sciences & Interventional Radiology, Sree Chitra Tirunal Institute for Medical Sciences and Technology, Medical College Campus, Medical College, Thiruvananthapuram, Kerala, 695011, India; 3 Department of Neurology, Sree Chitra Tirunal Institute for Medical Sciences and Technology, Medical College Campus, Medical College, Thiruvananthapuram, Kerala, 695011, India

## Abstract

The spectrum of presentation of intracranial hypotension is clinically perplexing. We report a case of 31-year-old post-partum woman who underwent an uneventful caesarean section under spinal anesthesia. From the second day of surgery she developed postural headache, the headache lost its postural character after few days. She then developed seizures and ataxic hemiparesis. Magnetic resonance imaging showed features of severe intracranial hypotension in the brain and the spinal cord, and magnetic resonance venography showed cortical vein and partial superior sagittal sinus thrombosis. Prothrombotic (etiological) work-up showed Protein C and S deficiency. She responded to anticoagulation therapy and recovered completely. On review of literature two distinct groups could be identified obstetric and non-obstetric. The non-obstetric group included patients who underwent diagnostic lumbar puncture, intrathecal injection of medications and epidural anesthesia for non-obstetric surgeries. Poor outcome and mortality was noted in non-obstetric group, while obstetric group had an excellent recovery.

## Introduction

Post-lumbar puncture (PL) headache is common, occurring in 10–30% of patients due to persistent cerebrospinal fluid (CSF) leakage after a lumbar puncture performed for anesthesia or diagnostic purposes ^1^. Post-lumbar puncture intracranial hypotension syndrome (PL-IHS) with persistent headache and neurological deficits is rare. PL-IHS is observed in multiple settings post-diagnostic lumbar puncture ^2^, post-spinal anesthesia/analgesia for abdominal and lower-limb surgeries ^3^, post-epidural anesthesia/analgesia during labor for pain and caesarean section ^4^, and post-intrathecal injection for chemotherapy and post-myelography ^5^. Usually it has a benign course, with most patients making complete recovery with or without an epidural blood patch (EBP). Rarely complications may develop in the form of subdural hygroma ^6^ and neurological deficits due to brainstem compression ^7^. We present a case with PL headache in a post-partum female progressing to cerebral venous thrombosis (CVT). We reviewed the literature to identify predictors of outcome.

## Case report

A 31-yr-old female with pregnancy-induced hypertension (PIH) underwent caesarean section (CS) at 35 weeks of pregnancy (due to fetal growth retardation). Spinal anesthesia was used for the procedure. On the second day post-partum, she noticed diffuse occipital headache and neck pain when she tried to get off the bed, which improved on lying down. This postural headache was persistent over next 8 days following which it changed character and became continuous. A diagnosis of PL headache was made and she was discharged on Ibuprofen 400mg thrice daily. On 12 ^th^ post-partum day she had a generalized tonic clonic seizure and had persistent drowsiness. She was taken to local hospital for seizure; there she received lorazepam 4mg slow IV bolus followed by phenytoin 900mg IV infusion over 60 minutes. After stabilization of vitals she was transferred to our comprehensive stroke care center. Prior to this episode she had been detected to have PIH in two earlier pregnancies and the first pregnancy had resulted in intrauterine death. During the present pregnancy she received dalteparin 5000 IU/day subcutaneous injection from 6 weeks pregnancy from local maternity centre with suspicion of anti-phospholipid syndrome; it was continued until the 8 ^th^ post-partum day. There was no past history of abortions or deep vein thrombosis.

On examination in the emergency room after 12 hours of seizure she was conscious and oriented. She had no papilledema and her visual acuity and visual field were normal. She had weakness and appendicular ataxia of the left upper limb and mild dysarthria. Her National Institute of Health Stroke Scale on admission was 3.

**Figure 1.**
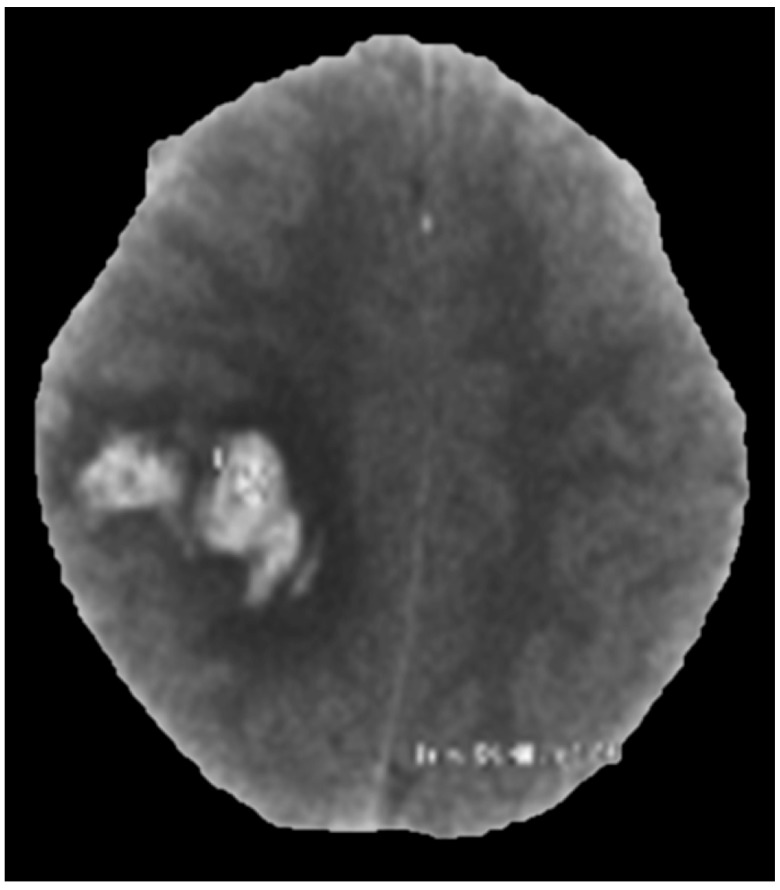
CT scan head showing a right high parietal hemorrhagic infarct with surrounding edema.

**Figure 2.**
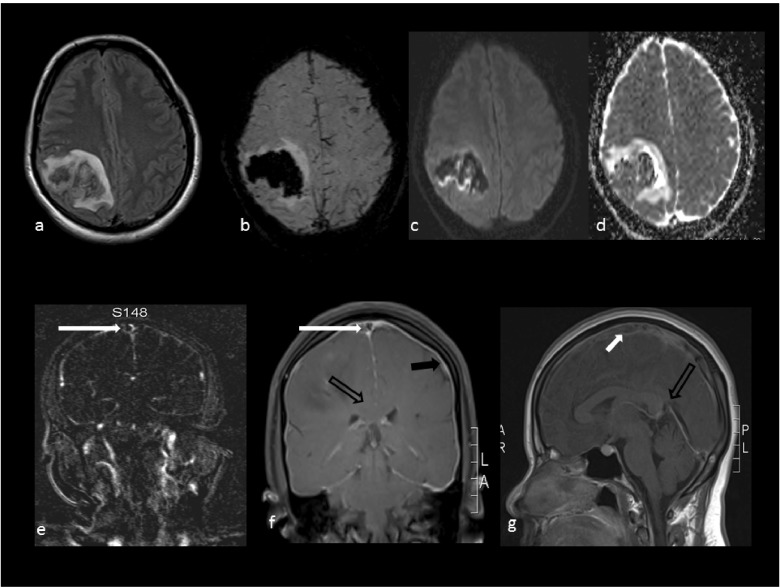
MR imaging. **a**-Flair, **b**-SWI, **c**-DWI map, **d**-ADC map show right high parietal hematoma without diffusion restriction; **e**-MR venogram shows thrombus in the sagittal sinus thrombus (white solid arrow); **f**-post-contrast T1W coronal sequence shows enhancement of the pachymeninges (black solid arrow), sagging of the corpus callosum (black open arrow) and filling defect of the sagittal sinus (white solid arrow); **g**-post-contrast T1W sagittal sequence shows thrombus in the sagittal sinus (white solid arrow) and narrowing of angle between the vein of Galen and straight sinus (black open arrow).

**Figure 3.**
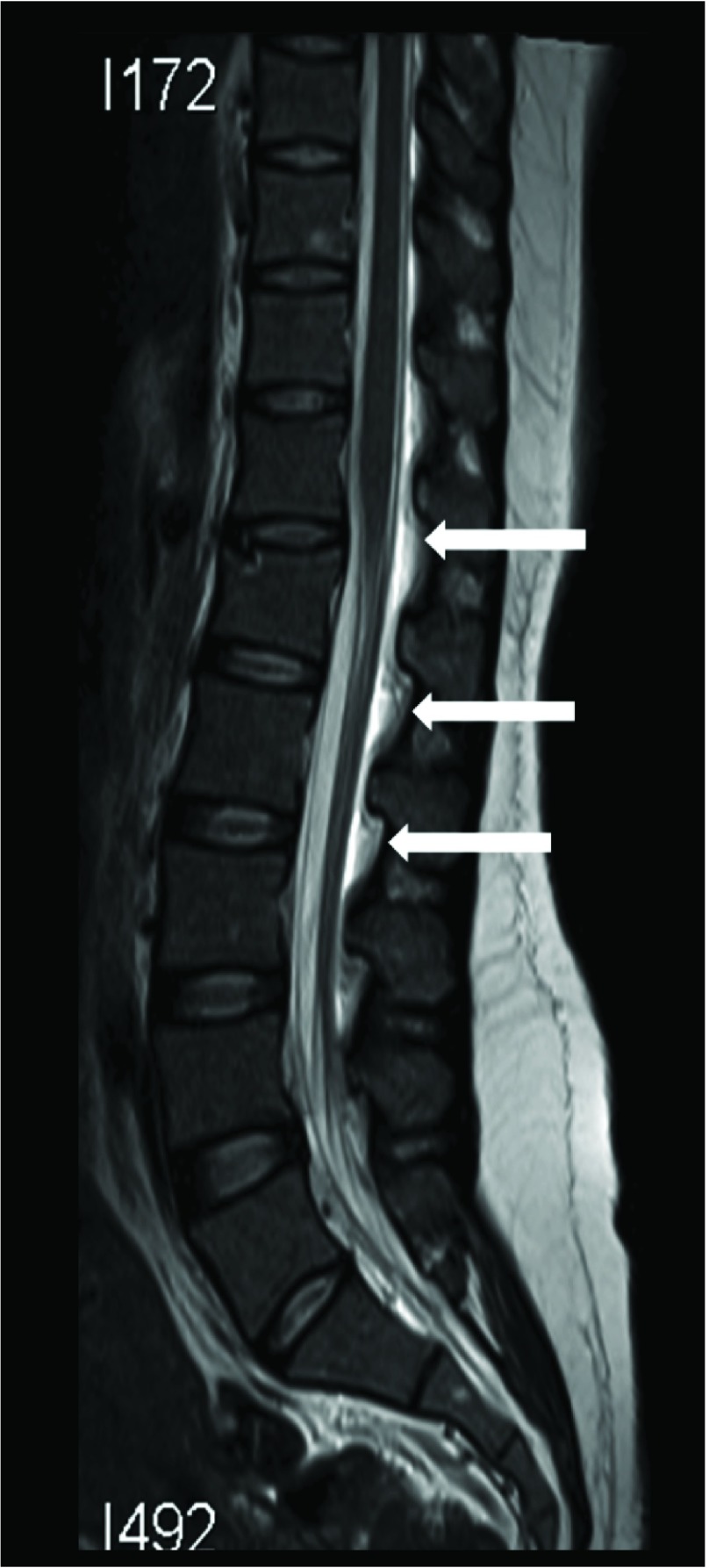
MR imaging of the spine. T2W sequence shows rim epidural CSF collection in the posterior aspect D11–L3 vertebra suggestive of CSF leak (white solid arrow).

She was investigated with a CT scan of the head ( Figure 1) after 8 hours of ictus at a local hospital; this showed a right high parietal hemorrhagic infarct. MR imaging of the brain ( Figure 2a–g) and spine ( Figure 3) (sequences: T1 Weighted, T2 Weighted, fluid attenuated inversion recovery (FLAIR), susceptibility weighted imaging (SWI), diffusion weighted imaging (DWI), apparent diffusion coefficient (ADC) and MR venography (MRV) was done after 14 hours of seizure ictus. This showed a right posterior high parietal lobe hematoma with mass effect and right side superficial cortical veins and partial sagittal sinus thrombosis. In addition there was evidence of intracranial hypotension with CSF leakage at the lumbar puncture site, as evidenced by corpus callosum sagging, pachymeningeal enhancement, and diffuse prominence of the cortical vein and rim of CSF seen in the epidural space from the D10 to the L3 vertebra. Her routine blood investigations, including a haemogram, liver function test and renal function test, were within normal limits. Erythrocyte sedimentation rate was elevated with 40 mm/hour. Her coagulation parameters including prothrombin time and antithromboplastin time were normal. Prothrombotic (etiological) work-up sent prior to starting of heparin therapy revealed reduced Protein C and Protein S activity of 31% (normal range 67–195%, plasma-clotting time based assay) and 26% (normal range 55–123%, plasma-clotting time based assay), respectively. Antithrombin III antigen level was normal at 209 mg/l (normal range: 170–300 mg/l, chromogenic assay). Factor V Leiden mutation (real time PCR method) was not detected. The vasculitic work up (antineutrophil antibody, ANA, double stranded DNA, dsDNA, antiphospholipid antibody, APLA, IgG and IgM and antineutrophil cytoplasmic antibody, ANCA) was negative.

Unfractionated heparin was started (16 hours after time of onset) at 800 units/hour to maintain plasma thromboplastin time between 75–90 seconds along with bed rest and caffeine immediately after the MRI venography. She responded early and was discharged after 6 days on warfarin 4mg once daily and optimized international normalized ratio (INR) of 2.1. On discharge she did not have any headache and neurological deficit had subsided, with NIHSS of 0 and modified Rankin Scale (mRS) of 0. On follow-up after 3 months, she had no further recurrences or new symptoms.

## Discussion

We describe a case of PL-IHS as suggested by the typical postural headache of the patient and the change of pattern of headache, which could have been the time of transition when cortical venous thrombosis developed. The brain and spine features seen on MR imaging are also supportive of this diagnosis, showing classical features of intracranial hypotension and cortical venous thrombosis. Our patient had multiple predisposing conditions, such as the early post-partum period, a history of previous intrauterine death and reduced activity of Protein C and Protein S. These predisposing conditions increased the likelihood of occurrence of thrombosis in our patient with PL-IHS. Similar findings have been also described by Wilder-Smith *et al.* ^3^; of their five PL-IHS patients, three had a predisposing prothrombotic condition.

The pathogenesis of the PL-IHS-induced CVT can be explained by the Monro-Kellie-Abercrombie doctrine. This suggests that the skull is a rigid structure, and the brain volume, venous blood and CSF are in state of equilibrium, so reduction or increase of either element leads alteration in the volume of the other two. In IHS, the CSF volume and pressure are reduced significantly. Two changes occur as a result of this: first in the venous compartment, there is increase in the intracranial venous volume, and second there is descent of the brain and brainstem structures. The venous volume expansion is due to the venous stasis and dilation of the sinuses, cortical and spinal veins. This change occurs first in the meninges, both pachymeninges and leptomeninges. The pachymeninges do not have a blood-brain barrier; this leads to contrast extravasation and hence the post-contrast enhancement. Further, due to the descent of the brain there is distortion and stretching of the veins. All the above changes are further aggravated in erect posture when there is acute dilation of the veins and further stretch on the venous walls, which leads to postural headache. This along with venous stasis may lead to CVT in some patients. This hypothesis has been further bolstered by the study by Canhao *et al.* ^8^ who showed reduction in the velocity of blood flow by approximately 50% in the straight sinus in patients after lumbar puncture.

## Review of literature

On reviewing the published literature for PL-IHS followed by CVT, we were able to extract 52 cases from search on PubMed on September 2013 (Data Set 1). These patients could be divided into two major groups ( Table 1): an obstetric associated PL-IHS group and non-obstetric associated group, including post-diagnostic lumbar puncture spinal or epidural anesthesia for non-obstetric surgeries, lumbar intrathecal injection for chemotherapy or myelography, and insertion of lumbar drain.

**Table 1.** Patient characteristics of the post-lumbar puncture intracranial hypotension and cerebral venous thrombosis (literature review).

Characteristic	Obstetric group (n=18)	Non-obstetric group (n=36)	
		Diagnostic LP (n=18)	Surgery and intrathecal injection (n=18)
Age years, mean (SD)	27.1±6.36	31.54±9.13	35.5±10.76
Duration of symptoms (days)	4–12	2–9	2–11
Postural headache	13/18 (72.2%)	9/9 (100%)	9/10 (90%)
Change in pattern of headache	8/17 (47.1%)	7/9 (77.8%)	4/10 (40%)
Co-morbidities	Pro-thrombotic condition (6/17) (35.3%)	Demyelinating disorders (14/17) (82.4%)	Pro-thrombotic condition (6/9) (66.7%)
Use of prophylactic heparin prior to CVT	2/16	None	2/16
Use of EBP prior to CVT	8/17 (47.1%)	1/9 (11.1%)	None
Sinus/vein involvement	Cortical vein 9/16 (56.2%) SSS 8/16 (50%)	SSS-7/10 (70%)	SSS 4/8 (50%) Cortical vein 4/8 (50%)
Treatment given	Anticoagulation 12 EBP 1 Combined 1 None 2 Not known 2	Anticoagulation 8/9 Combined 1/9	Anticoagulation 8 EBP 1
Poor response to therapy after CVT	None	3/12 (25%)	3/11 (27.2%)
Mortality	None	1	1

Review of literature of cerebral venous thrombosis associated with post-lumbar puncture intracranial hypotension This table shows 52 cases identified by reviewing the published literature (PubMed search) in September 2013. Details include obstetric associated PL-IHS patients and non-obstetric associated patients, subjected to post-diagnostic lumbar puncture spinal or epidural anesthesia for non-obstretic surgeries, lumbar intrathecal injection for chemotherapy or myelography, and insertion lumbar drain.

### Obstetric associated group

The CVT symptoms developed after 4–12 days of the lumbar puncture. Approximately three quarters of the patients had postural headache at the onset and a change in the pattern of headache was observed in approximately half of the patients with the onset of CVT symptoms. This was the headache becoming continuous, becoming diffuse or losing its postural character. These patients had prothrombotic predisposition in 35% of cases or prior history of oral contraceptive pill intake as additional pathogenetic factors. The prothrombotic conditions were Protein C and S deficiency ^9– 11^, heterozygous factor V Leiden mutation ^12^ and prothrombin mutation 20210A ^13^. Patients in this group had uniformly good response with complete resolution of symptoms in less than 4 weeks. Neither prior epidural blood patch ^11, 12^ nor prophylactic heparin were able to prevent occurrence of CVT in post-partum PL-IHS.

### Non-obstetric group

a) Post-diagnostic lumbar puncture (n=17)

The majority of the patients (82.5%) in this group had demyelinating disorder and had received intravenous high dose corticosteroids (IVCS) within a few days, such as multiple sclerosis ^2^, recurrent optic neuritis ^3^ and probable Neuromyelitis optica ^14^. None of these patients had any prothrombotic condition as observed in the Obstetric associated Group. All patients uniformly had postural headache at the onset and this was followed by a change in the pattern of headache as described above in 77.8% of patients with the development of CVT. Superior sagittal sinus was involved in 70% of the cases. One-quarter of the patients in this group were reported to have had a poor outcome ^2, 15^. The underlying disease and sagittal sinus involvement may have been responsible for the poor outcome.

Reports of CVT in demyelinating disorders have increased in last decade ^2^ but the exact pathogenesis remains to be elucidated. The concomitant use of IVCS has been proposed to be causative factor for the CVT. This can partly be explained by the role of high dose steroids on CSF dynamics and vascular endothelium: steroid treatment has a paradoxical effect on CSF absorption and can either impair or facilitate its absorption. Steroids have a vasodilatory effect on the vessel; this could further aggravate the venous stasis present in the PL-IHS. We hypothesize that the combined changes in CSF absorption and increased venous stasis in patients with lower intracranial hypotension may increase the likelihood of the venous thrombosis inpatient receiving IVCS.

b) Other procedures group

The average age of at presentation was 35.5±10.76 years. This group was heterogeneous, including patients that had experienced post-epidural steroid injection ^16, 17^, post-intrathecal methotrexate ^5^, post-myelography ^3^, post-epidural anesthesia ^3^, post-spinal anesthesia ^18^ and post-lumbar drain ^19^. Underlying prothrombotic conditions were Factor V Leiden mutation ^3^, Protein C deficiency ^17^ and lymphocytic lymphoma ^5^. In other cases either etiological work-up was non-revealing or was not done (Data Set 1). Poor outcome was noted in 27.2% (3/11) patients and one patient died during the illness ^3^. Apart from the age, post-partum state and outcome this group had similar characteristics and risk factors as obstetric associated group.

Since its initial description by Schou and Scherb in 1986 of PL-IHS and CVT ^18^, there has been the ongoing debate about whether they are both mere associations and whether their occurrence is only coincidence. But over the last two decades there is formidable evidence suggesting causality ^8^, that is, of PL-IHS causing CVT, though the exact pathogenetic mechanism remains to be conclusively demonstrated. Further, the various settings in which the phenomenon has been observed suggest multiple risk factors. Over the last 25 years of its description, the clinical characteristics have consistently demonstrated the headache pattern, starting as postural headache and then with a change in the headache character as it loses its orthostatic character; this should alert the treating clinician that the patient may have developed CVT. Though the phenomenon is very rare compared to the number of lumbar punctures being performed, the morbid outcome in one-quarter of patients reported should caution us. The propensity for PL-IHS is increased in patients with prothrombotic conditions; prophylactic heparin may prevent the occurrence of the CVT in this group of patients. This generates questions regarding the dose and duration of the heparin, type of heparin to be used, assessment measures etc, which further studies can answer. Patients with known prothrombotic conditions who undergo lumbar puncture for various procedures should be aggressively managed, including hydration and supine posture, and they may benefit from heparin prophylaxis.

## Data availability

Figshare: Review of literature of cerebral venous thrombosis associated with post-lumbar puncture intracranial hypotension, http://dx.doi.org/10.6084/m9.figshare.920192 ^20^

## Consent

Written informed consent for publication of clinical details and clinical images was obtained from the patient on Institutional format.
