# Supplementary material for: Cerebral venous thrombosis in post-lumbar puncture intracranial hypotension: case report and review of literature
Source: F1000Res. 2014 Feb 11;3:41. [Version 1] doi: 10.12688/f1000research.3-41.v1 (PMC3945949; doi:10.12688/f1000research.3-41.v1)
Supplement: Peer review history

# Peer review: Referee response for version 1

**Published:** 2014-02-17

**Authors:** J David Spence

**Affiliations:** 1 Robarts Research Institute, University of Western Ontario, London, ON, Canada

This case report and review of the literature may be helpful to the patients of physicians who are not aware of this syndrome.

I have read this submission. I believe that I have an appropriate level of expertise to confirm that it is of an acceptable scientific standard.

# Peer review: Referee response for version 1

**Published:** 2014-02-12

**Authors:** Bruce Campbell

**Affiliations:** 1 Department of Neurology, Royal Melbourne Hospital, Melbourne, VIC, Australia

An interesting case with a well conducted review of similar cases in the literature. It is a good clinical teaching point to consider dural sinus thrombosis if there is a change in pattern of post-lumbar puncture headache.

I have read this submission. I believe that I have an appropriate level of expertise to confirm that it is of an acceptable scientific standard.
